# Supplementary material for: Lysosomal Dysfunction: Connecting the Dots in the Landscape of Human Diseases
Source: Biology (Basel). 2024 Jan 7;13(1):34. doi: 10.3390/biology13010034 (PMC10813815; doi:10.3390/biology13010034)
Supplement: Supplementary file 1 [file biology-13-00034-s001.zip › biology-2755159-supplementary.pdf]

Gene	Disease	Position (GRCh38.p12)	Change	Type	dbSNP rsid	Ref
GALNS	MPS IVA (Morquio A syndrome)	chr16:88841948	G>A	Missense	rs1028668536	[73]
		chr16:88841077	T>A	Missense	rs118204438	[118]
		chr16:88835794	C>T	Stop Gained	rs118204449	[89]
		chr16:88837646	T>C	Missense	rs786205899	[117]
		chr16:88836222	G>C	Missense	rs118204435	[97]
		chr16:88841936	G>A / G>C	Missense	rs118204441	[67]
		chr16:88835335	C>T	Missense	rs118204442	[51]
		chr16:88832065	G>C	Missense	rs118204446	[104]
		chr16:88842772	C>T	Missense	rs118204447	[89]
		chr16:88835240	C>A / C>T	Missense	rs118204448	[89]
		chr16:88826822	C>T	Missense	rs267606838	[88]
GLB1	MPS IVB (Morquio B syndrome)	chr3:33014263	C>A	Missense	rs72555363	[64]
		chr3:33014280	insCT	Frameshift	rs1553606128	[111]
		chr3:33051951-33051952	delG	Frameshift	rs1553610382	[76]
		chr3:33053518	C>G	Missense	rs1553610553	[47]
		chr3:33068968	T>C	Missense	rs1553612220	[48]
		chr3:33058220	C>T	Missense	rs189115557	[56]
		chr3:33068245	G>A / G>T	Missense	rs192732174	[121]
		chr3:33014213-33014218	delC / dupC	Frameshift	rs794729217	[128]
		chr3:32997311	G>A / G>T	Missense	rs794727165	[129]
		chr3:33068241	G>A	Missense	rs778700089	[54]
		chr3:33072543	C>G / C>T	Splice Donor	rs778423653	[62]
HYAL1	MPS IX	chr3:50302155	C>T	Missense	rs104893743	[110]
IDUA	MPS type I (Hurler syndrome)	chr4:1002747	G>A	Stop Gained	rs121965019	[114]
		chr4:1002117	C>T	Stop Gained	rs121965023	[87]
		chr4:1004292	C>G / C>T	Stop Gained	rs121965025	[92]
		chr4:1002087	G>A	Missense	rs121965030	[87]
		chr4:1002333	T>G	Missense	rs121965033	[65]

Gene	Disease	Position (GRCh38.p12)	Change	Type	dbSNP rsid	Ref
IDUA (Cont.)		chr4:1002275	G>C	Missense	rs199801029	[61]
		chr4:1004391	T>G	Stop Lost	rs387906504	[58]
		chr4:1004083	delC	Stop Gained	rs398123258	[85]
		chr4:1001672	G>A / G>C	Intron	rs762411583	[69]
		chr4:1002325	C>A / C>G	Stop Gained	rs764196171	[69]
		chr4:1001701-1001706	dupTGCTC	Frameshift	rs786200915	[91]
		chr4:1001742	T>C	Missense	rs869025584	[90]
		chr4:1002747	G>A	Stop Gained	rs121965019	[114]
		chr4:1003418	C>G / C>T	Missense	rs121965021	[108]
		chr4:1002392	A>C	Missense	rs121965024	[78]
		chr4:1003108	G>A / G>C	Missense	rs121965026	[58]
		chr4:1003102	T>C	Missense	rs121965027	[58]
		chr4:1004286	C>A / C>G / C>T	Stop Gained	rs121965031	[93]
		chr4:1002333	T>G	Missense	rs121965033	[65]
		chr4:1003418	C>G / C>T	Missense	rs121965021	[65]
IDS	MPS type II (Hunter syndrome)	chrX:149482996	C>A / C>G / C>T	Missense	rs113993946	[102]
		chrX:149498308	C>G / C>T	Splice Acceptor	rs113993947	[130]
		chrX:149482974	C>T	Stop Gained	rs199422230	[103]
		chrX:149498305-149498307	delGT	Frameshift	rs483352904	[103]
		chrX:149503379-149503382	delGAG	Inframe Deletion	rs483352905	[53]
		chrX:149504189-149504191	dupG	Frameshift	rs797044671	[82]
		chrX:149500992-149500993	delAAinsCCAGCTATACGG	Frameshift	rs869025304	[130]
		chrX:149496458-149496474	delACCTCGGGATCGGG	Stop Gained	rs869025305	[130]
		chrX:149486972-149486975	delAA	Frameshift	rs869025306	[130]
		chrX:149482936	delA	Frameshift	rs869025307	[130]
		chrX:149490313	C>A	Splice Donor	rs869025308	[130]
		chrX:149500977	G>C / G>T	Missense	rs104894856	[52]
		chrX:149503326	T>C	Missense	rs104894861	[103]

Gene	Disease	Position (GRCh38.p12)	Change	Type	dbSNP rsid	Ref
IDS (cont.)		chrX:149482894	C>G	Missense	rs199422228	[52]
		chrX:149483135	A>C	Missense	rs199422229	[103]
		chrX:149482997	G>A	Missense	rs199422231	[49]
SGSH	MPS type IIIA (Sanfilippo syndrome type A)	chr17:80213815	C>A / C>T	Missense	rs104894635	[63]
		chr17:80217084	G>A / G>C	Missense	rs104894637	[55]
		chr17:80213876	A>G	Missense	rs1057521801	[74]
		chr17:80212128	A>G	Missense	rs138504221	[119]
		chr17:80213852	G>A / G>C	Stop Gained	rs374621913	[74]
		chr17:80210881-80210882	delG	Frameshift	rs770947426	[85]
		chr17:80210826	delC	Frameshift	rs777956287	[84]
		chr17:80210934-80210935	dupG	Frameshift	rs778700037	[85]
		chr17:80214672	C>T	Missense	rs104894638	[101]
		chr17:80210856	C>A / C>G / C>T	Stop Gained	rs104894640	[46]
		chr17:80214738	G>A	Missense	rs104894642	[46]
		chr17:80214218	C>G / C>T	Missense	rs104894643	[44]
		chr17:80217061	G>A	Missense	rs104894636	[66]
		chr17:80210663	C>T	Missense	rs104894641	[46]
		chr17:80212128	A>G	Missense	rs138504221	[94]
		chr17:80213815	C>A / C>T	Missense	rs104894635	[68]
NAGLU	MPS type IIIB (Sanfilippo syndrome type B)	chr17:42543882	C>G / C>T	Stop Gained	rs104894591	[83]
		chr17:42541074	C>T	Stop Gained	rs104894592	[122]
		chr17:42543699	C>T	Missense	rs104894597	[60]
		chr17:42543700	G>A / G>C / G>T	Missense	rs104894598	[95]
		chr17:42541127	C>G	Missense	rs118204025	[81]
		chr17:42543997	C>T	Missense	rs746006696	[45]
		chr17:42537494	delT	Frameshift	rs886039894	[50]
		chr17:42543950	dupG	Frameshift	rs886039895	[50]
		chr17:42536414	T>C	Missense	rs118204024	[96]

Gene	Disease	Position (GRCh38.p12)	Change	Type	dbSNP rsid	Ref
NAGLU (Cont.)		chr17:42544027	G>A / G>T	Missense	rs104894590	[83]
		chr17:42543934	G>A	Missense	rs104894593	[83]
		chr17:42543933	C>A / C>T	Missense	rs104894594	[127]
		chr17:42543568	C>T	Missense	rs104894595	[127]
		chr17:42543699	C>T	Missense	rs104894597	[127]
		chr17:42543700	G>A / G>C / G>T	Missense	rs104894598	[127]
		chr17:42537433	A>G	Missense	rs753520553	[123]
		chr17:42543214	T>C	Missense	rs796052122	[45]
		chr17:42538691	C>T	Missense	rs104894601	[79]
HGSNAT	MPS type IIIC (Sanfilippo syndrome type C)	chr8:43173740	C>T	Missense	rs121908282	[112]
		chr8:43178184	T>G	Stop Gained	rs121908283	[112]
		chr8:43193824	T>A	Missense	rs121908284	[112]
		chr8:43182162	C>T	Missense	rs121908285	[124]
		chr8:43197682	C>T	Missense	rs121908286	[80]
		chr8:43159045	G>A	Splice Donor	rs193066451	[70]
		chr8:43158921	A>G	Splice Acceptor	rs483352896	[80]
		chr8:43193790	G>A	Missense	rs753355844	[120]
		chr8:43158710	A>T	Missense	rs754875934	[115]
		chr8:43191554	G>T	Missense	rs764206492	[112]
		chr8:43158949	G>C	Missense	rs797045120	[115]
GNS	MPS type IIID (Sanfilippo syndrome	chr12:64737039	G>A	Stop Gained	rs119461974	[59]
		chr12:64728988	G>A	Stop Gained	rs119461975	[126]
		chr12:64728987	delT	Frameshift	rs483352898	[125]
		chr12:64729017-64729018	insAGGAC	Frameshift	rs483352899	[126]
		chr12:64723088	dupC	Frameshift	rs483352900	[116]
ARSB	MPS type VI (Maroteaux-Lamy syndrome)	chr5:78969095	C>A / C>T	Missense	rs118203938	[100]
		chr5:78969156	A>G	Missense	rs118203939	[98]
		chr5:78955486	A>G	Missense	rs118203940	[98]

Gene	Disease	Position (GRCh38.p12)	Change	Type	dbSNP rsid	Ref
ARSB (Cont.)		chr5:78985011-78985014	delC	Frameshift	rs431905493	[113]
		chr5:78839427	C>G	Splice Acceptor	rs431905495	[86]
		chr5:78839434	A>C	Intron	rs431905496	[71]
		chr5:78984965	C>G / C>T	Missense	rs118203942	[72]
		chr5:78839391	T>C / T>G	Missense	rs118203944	[72]
		chr5:78964477	T>C	Missense	rs118203943	[72]
GUSB	MPS type VII (Sly syndrome)	chr7:65967863	C>T	Stop Gained	rs121918179	[57]
		chr7:65979782	G>A	Missense	rs121918181	[109]
		chr7:65974701	G>A	Stop Gained	rs121918185	[106]
		chr7:65967767	G>A	Synonymous	rs377519272	[57]
		chr7:65973676-65973677	delGA	Intron	rs786200863	[75]
		chr7:65974348	C>T	Stop Gained	rs121918180	[75]
		chr7:65974626	G>A	Missense	rs121918173	[99]
		chr7:65979477	G>A	Missense	rs121918174	[77]
		chr7:65974923	G>A	Missense	rs121918175	[107]
		chr7:65974923	G>A	Missense	rs121918176	[107]
		chr7:65979866	G>A	Missense	rs121918177	[57]
		chr7:65967900	T>C	Missense	rs121918178	[57]
		chr7:65974934	C>G / C>T	Missense	rs121918182	[105]
		chr7:65964382	C>A	Missense	rs121918183	[105]
		chr7:65960972	C>A	Missense	rs121918184	[43]
		chr7:65960997	G>A	Missense	rs121918172	[99]

Supplementary table S1: Clinically relevant SNPs in Mucopolysaccharidoses.

Gene	Disease	Position (GRCh38.p12)	Change	Type	dbSNP rsid	Ref
GAA	Pompe disease	chr17:80113361-80113362	delC	Frameshift	rs1057516581	[149]
		chr17:80102793	A>C	Intron	rs12450199	[150]
		chr17:80110837	G>A	Stop Gained	rs140826989	[172]
		chr17:80113350	C>A / C>T	Missense	rs121907938	[154]
		chr17:80107818	G>A	Missense	rs121907945	[169]
		chr17:80107894	T>A / T>C	Missense	rs121907936	[172]
		chr17:80113242	G>A / G>C	Missense	rs1800309	[160]
		chr17:80112072	G>A	Missense	rs1800307	[166]
		chr17:80110726	G>C / G>T	Splice Acceptor	rs147804176	[172]
		chr17:80108512	T>C	Missense	rs1555600061	[172]
		chr17:80113309-80113310	CA>GG	Missense	rs1555601773	[149]
		chr17:80117016	G>A / G>C	Stop Gained	rs1800312	[172]
		chr17:80107616	C>T	Missense	rs200856561	[172]
		chr17:80112664	C>A / C>T	Missense	rs369531647	[171]
		chr17:80112922	C>A / C>T	Missense	rs28940868	[149]
		chr17:80104552	C>A / C>T	Intron	rs1055945806	[168]
		chr17:80108609	T>C	Splice Donor	rs1057516215	[157]
		chr17:80117005	C>T	Stop Gained	rs1057516277	[164]
		chr17:80108487	A>G	Splice Acceptor	rs1057516290	[155]
		chr17:80116992	G>A	Stop Gained	rs1057516328	[155]
		chr17:80112081	G>A / G>C	Missense	rs991082382	[174]
		chr17:80112680	C>G / C>T	Missense	rs914396317	[146]
		chr17:80110953	C>A / C>G / C>T	Missense	rs892129065	[148]
		chr17:80118210-80118213	delCA	Frameshift	rs886043343	[168]
		chr17:80110950	G>A	Missense	rs121907937	[162]
		chr17:80107837	T>C / T>G	Missense	rs121907940	[168]
		chr17:80110974-80110975	TC>GT	Missense	rs121907941	[147]
		chr17:80111023	C>T	Missense	rs121907942	[158]

Gene	Disease	Position (GRCh38.p12)	Change	Type	dbSNP rsid	Ref
GAA (Cont.)		chr17:80118271	C>T	Stop Gained	rs121907943	[161]
		chr17:80107574	C>A / C>T	Missense	rs121907944	[162]
		chr17:80118752	G>T	Missense	rs1221948995	[161]
		chr17:80119318	T>A	Missense	rs1245412108	[167]
		chr17:80117675	C>T	Stop Gained	rs1344266804	[159]
		chr17:80112643	G>A	Missense	rs1393386120	[163]
		chr17:80112892	C>A / C>T	Missense	rs1414146587	[175]
LAMP2	Danon disease	chrX:120449006	G>A	Stop Gained	rs104894857	[153]
		chrX:120442599	C>T	Missense	rs104894858	[156]
		chrX:120448978-120448980	delCTCinsTA	Frameshift	rs1060502302	[165]
		chrX:120449086	A>T	Stop Gained	rs137852527	[170]
		chrX:120442650	G>A / G>T	Stop Gained	rs727503118	[152]
		chrX:120446304	C>A / C>T	Splice Donor	rs727503119	[173]
		chrX:120456650	C>T	Splice Donor	rs727503120	[165]
		chrX:120441729	C>T	Splice Donor	rs727504742	[151]

Supplementary table S2: Clinically relevant SNPs in Glycogenoses

Gene	Disease	Position (GRCh38.p12)	Change	Type	dbSNP rsid	Ref
GLA	Fabry disease	chrX:101398467	C>A / C>T	Missense	rs104894828	[208]
		chrX:101403846	G>A	Missense	rs104894834	[208]
		chrX:101401752	C>A / C>G / C>T	Missense	rs104894845	[199]
		chrX:101398942	T>C	Missense	rs28935197	[198]
		chrX:101403843	A>G / A>T	Missense	rs869312142	[197]
GBA	Gaucher disease	chr1:155235252	A>C / A>G	Missense	rs421016	[195]
NPC1	Niemann-Pick Disease Type C	chr18:23552736	C>G / C>T	Intron	rs1055204017	[202]
		chr18:23560273-23560277	delA	Frameshift	rs1057518711	[212]
		chr18:23556527	G>A	Stop Gained	rs1474434210	[213]
		chr18:23539826	G>A	Missense	rs753768576	[213]
		chr18:23556358	C>T	Missense	rs139751448	[210]
		chr18:23539447	G>A	Missense	rs143124972	[210]
		chr18:23536811	G>A	Missense	rs28942104	[210]
		chr18:23538610-23538611	delCT	Frameshift	rs756815030	[200]
		chr18:23534475-23534476	delC	Frameshift	rs758231839	[194]
		chr18:23544987-23544990	delC	Frameshift	rs886042270	[202]
		chr18:23538564	G>C	Missense	rs80358257	[201]
		chr18:23538609	C>A / C>G / C>T	Missense	rs80358254	[201]
		chr18:23536736	A>G	Missense	rs80358259	[201]
		chr18:23538572	G>A	Missense	rs150334966	[207]
		chr18:23544402	G>A	Missense	rs1555634422	[211]
		chr18:23539878	C>T	Missense	rs768999208	[214]
		chr18:23536736	A>G	Missense	rs80358259	[196]
NPC2	Niemann-Pick Disease Type C	chr14:74486386	G>A	Stop Gained	rs80358262	[204]
		chr14:74486378	G>T	Stop Gained	rs80358263	[209]
		chr14:74484426	C>A / C>T	Stop Gained	rs80358266	[205]

Gene	Disease	Position (GRCh38.p12)	Change	Type	dbSNP rsid	Ref
NPC2 (Cont.)		chr14:74486324	C>T	Intron	rs80358268	[205]
		chr14:74484579	A>G	Missense	rs11694	[206]
		chr14:74486404	C>T	Missense	rs80358261	[206]
		chr14:74484483	A>G	Missense	rs80358264	[206]
		chr14:74484420	G>A	Missense	rs104894458	[203]

Supplementary table S3: Clinically relevant SNPs in Sphingolipidoses.

Gene	Disease	Position (GRCh38.p12)	Change	Type of variant	dbSNP rsid	Ref
CLN3	Neuronal Ceroid Lipofuscinosis Type 3	chr16:28482500	C>A / C>T	Stop Gained	rs121434286	[245]
		chr16:28486427	G>T	Stop Gained	rs267606737	[257]
		chr16:28487492-28487493	delC	Frameshift	rs386833720	[259]
		chr16:28486617	C>T	Missense	rs786201028	[253]
		chr16:28482500	C>A / C>T	Stop Gained	rs121434286	[252]
CLN6	Neuronal Ceroid Lipofuscinosis Type 6	chr15:68214373	C>A / C>G / C>T	Stop Gained	rs104894483	[248]
		chr15:68209639	G>A / G>C	Stop Gained	rs104894486	[260]
		chr15:68211289-68211294	delATA	Inframe Deletion	rs121908079	[248]
		chr15:68211698-68211705	delATG	Inframe Deletion	rs121908080	[255]
		chr15:68211845-68211850	dupG	Frameshift	rs397515352	[252]
		chr15:68211765-68211768	delAG	Frameshift	rs774543080	[247]
		chr15:68229578-68229579	delC	Frameshift	rs786205065	[247]
		chr15:68211258	C>A	Intron	rs786205066	[260]
		chr15:68214316-68214319	dupCGTT	Frameshift	rs786205067	[255]
		chr15:68211793	C>T	Missense	rs104894484	[247]
CLN8	Neuronal Ceroid Lipofuscinosis Type 8	chr8:1780316	C>T	Missense	rs104894060	[262]
		chr8:1771142	G>A / G>C / G>T	Missense	rs137852883	[254]
		chr8:1780495	G>C	Missense	rs28940569	[246]
CTSD	Neuronal Ceroid Lipofuscinosis Type 10	chr11:1754969	dupT	Stop Gained	rs786205105	[243]
		chr11:1758994	C>A	Missense	rs797045137	[241]
		chr11:1753546	C>T	Missense	rs797045138	[241]
		chr11:1757343	A>T	Missense	rs121912789	[242]
		chr11:1753593	C>G	Missense	rs121912790	[242]

Gene	Disease	Position (GRCh38.p12)	Change	Type of variant	dbSNP rsid	Ref
CTSF	Neuronal Ceroid Lipofuscinosis Type 13	chr11:66565841	delG	Frameshift	rs753084727	[244]
		chr11:66568273	C>G	Splice Donor	rs797045136	[258]
		chr11:66566320	T>C	Missense	rs143889283	[244]
		chr11:66565833	T>C	Missense	rs397514731	[244]
MFSD8	Neuronal Ceroid Lipofuscinosis Type 7	chr4:127930787	A>C / A>G	Stop Gained	rs118203977	[256]
		chr4:127938781	A>T	Splice Donor	rs587778809	[256]
		chr4:127920743	G>A	Stop Gained	rs724159971	[250]
		chr4:127932981-127932982	insA	Intron	rs727502801	[250]
		chr4:127930752	C>T	Missense	rs118203975	[250]
		chr4:127921588	C>T	Missense	rs118203976	[249]
		chr4:127930800	G>A / G>T	Missense	rs140948465	[251]
		chr4:127921639	G>A	Missense	rs267607235	[261]
		chr4:127943775	C>A / C>T	Missense	rs749704755	[245]

Supplementary table S4: Clinically relevant SNPs in Neuronal Ceroid-Lipofuscinoses.

Gene	Disease	Position (GRCh38.p12)	Change	Genetic variant	dbSNP rsid	Ref
CTSA	Galactosialidosis	chr20:45898068	T>G	Missense	rs137854540	[289]
		chr20:45894040	T>A	Missense	rs137854544	[289]
		chr20:45894002	T>C	Missense	rs137854546	[289]
		chr20:45898065	G>A	Missense	rs137854547	[289]
		chr20:45897769	T>A / T>C	Missense	rs137854548	[289]
		chr20:45898107	A>G	Missense	rs137854549	[286]
		chr20:45893314	A>G	Intron	rs786200859	[279]
		chr20:45894704-45894706	delAT	Frameshift	rs875989777	[281]
		chr20:45894040	T>A	Missense	rs137854544	[281]
		chr20:45897736	A>G	Missense	rs137854543	[285]
FUCA1	Fucosidosis	chr1:23845837	G>A	Stop Gained	rs118204450	[288]
		chr1:23848671	C>A / C>G	Stop Gained	rs80358195	[287]
		chr1:23868043	G>A	Stop Gained	rs80358196	[284]
		chr1:23848649	C>T	Stop Gained	rs80358197	[284]
		chr1:23863148	G>A / G>T	Stop Gained	rs80358198	[284]
MAN2B1	Alpha-mannosidosis	chr19:12649418	G>A	Stop Gained	rs121434331	[278]
		chr19:12652376	G>A	Stop Gained	rs121434332	[278]
		chr19:12657482	G>A / G>C / G>T	Stop Gained	rs775200333	[280]
		chr19:12655693	C>G	Splice Donor	rs80338677	[277]
		chr19:12652462	T>C	Splice Acceptor	rs80338678	[276]
		chr19:12650103	C>T	Splice Donor	rs80338679	[276]
		chr19:12649932	G>A / G>C / G>T	Missense	rs80338680	[277]
		chr19:12649146	A>G	Missense	rs80338681	[282]
MANBA	Beta-mannosidosis	chr4:102657873	A>G	Missense	rs121434334	[283]
		chr4:102726614	C>A / C>T	Stop Gained	rs121434335	[275]
		chr4:102669004	G>A / G>C	Stop Gained	rs121434336	[275]

Supplementary table S5: Clinically relevant SNPs in Glycoproteinoses.
